# The evolution of parasite virulence under targeted culling and harvesting in wildlife and livestock

**DOI:** 10.1111/eva.13594

**Published:** 2023-09-28

**Authors:** Xander O'Neill, Andy White, Mike Boots

**Affiliations:** ^1^ Department of Mathematics Maxwell Institute for Mathematical Sciences, Heriot‐Watt University Edinburgh UK; ^2^ Department of Integrative Biology University of California Berkeley California USA; ^3^ Centre for Ecology and Conservation, Biosciences University of Exeter Cornwall UK

**Keywords:** disease management, disease transmission, mathematical model, virulence evolution

## Abstract

There is a clear need to understand the effect of human intervention on the evolution of infectious disease. In particular, culling and harvesting of both wildlife and managed livestock populations are carried out in a wide range of management practices, and they have the potential to impact the evolution of a broad range of disease characteristics. Applying eco‐evolutionary theory we show that once culling/harvesting becomes targeted on specific disease classes, the established result that culling selects for higher virulence is only found when sufficient infected individuals are culled. If susceptible or recovered individuals are targeted, selection for lower virulence can occur. An important implication of this result is that when culling to eradicate an infectious disease from a population, while it is optimal to target infected individuals, the consequent evolution can increase the basic reproductive ratio of the infection, $R_{0}$, and make parasite eradication more difficult. We show that increases in evolved virulence due to the culling of infected individuals can lead to excess population decline when sustainably harvesting a population. In contrast, culling susceptible or recovered individuals can select for decreased virulence and a reduction in population decline through culling. The implications to the evolution of virulence are typically the same in wildlife populations, that are regulated by the parasite, and livestock populations, that have a constant population size where restocking balances the losses due to mortality. However, the well‐known result that vertical transmission selects for lower virulence and transmission in wildlife populations is less marked in livestock populations for parasites that convey long‐term immunity since restocking can enhance the density of the immune class. Our work emphasizes the importance of understanding the evolutionary consequences of intervention strategies and the different ecological feedbacks that can occur in wildlife and livestock populations.

## 1 INTRODUCTION

Experimental, field and in particular theoretical studies exploring the evolution of parasite virulence and transmission are well‐developed (see Cressler et al., 2016 for a review). Theoretical studies have generally assumed that the evolution of parasite virulence (defined in this literature as an increased death rate due to infection) is dependent on a trade‐off with other host and parasite characteristics (Alizon et al., 2009; Anderson & May, 1982; Ewald, 1983). Typically it is assumed that a parasite cannot increase transmissibility without paying a cost in terms of increased virulence (the cost to the parasite of higher virulence is a reduced infectious period) (Anderson & May, 1982) although, other trade‐offs, including a virulence/recovery trade‐off have also been considered (Alizon, 2008; Anderson & May, 1983; Cressler et al., 2016; Frank, 1996). Within this extensive literature, theoretical models have been used successfully to explore the impact of different processes on the evolved level of virulence, including, for example, the role of host life‐history traits, infection transmission modes, parasite control strategies and spatial structure on the evolution of parasite traits (Ashby et al., 2019; Boots et al., 2014; Boots & Sasaki, 1999; Cressler et al., 2016; Jones et al., 2011). However, we only have a limited understanding of the evolution of virulence in response to culling/harvesting (Bolzoni & De Leo, 2013; Rozins & Day, 2017; Shim & Galvani, 2009). Moreover, the general theory has concentrated on evolution in wildlife host populations, that are regulated by a virulent parasite (see Cressler et al., 2016). However, given that ecological/epidemiological dynamics can feed back on to the evolution of the parasite there is the potential that the evolutionary outcomes in managed livestock populations, where mortality can be balanced by restocking to maintain a constant population size, may be significantly different to those in wildlife populations, particularly as infected individuals may be imported into populations in livestock systems.

Culling, in a broad context, is used to harvest wildlife/livestock populations or to control the density of overabundant wildlife species (Caughley & Sinclair, 1994; Fryxell et al., 2014). In a livestock system culling can be defined as the removal of undesirable animals from the herd to facilitate improvement in herd performance or to keep the herd size constant (Compton et al., 2017; Wakchaure et al., 2015). It may occur for a variety of reasons, such as age, health status, disposition, reproductive performance, milk production, inferior genetics or due to current economic factors (Bascom & Young, 1998; Hersom et al., 2018). Culling and mortality is inevitable in livestock systems farmed for meat and dairy production as individuals must exit the herd for slaughter, sale or die on‐farm (Compton et al., 2017). It has been suggested that culling rates may be greater than 30% per year (Haine et al., 2017), and while rates may vary at different times of the year, culling and replacement are ongoing processes and integral parts of herd management (Wakchaure et al., 2015). Culling of wildlife and livestock populations is also used as a management strategy to control infectious disease (Donnelly et al., 2006; Miguel et al., 2020; te Beest et al., 2011; Tildesley et al., 2009). In particular, reducing the population density below a threshold that supports the infection may lead to parasite extinction (Anderson et al., 1981; Lloyd‐Smith et al., 2005). However, it may also lead to compensatory population growth when culling is used to control highly virulent parasites (Tanner et al., 2019). Since any form of culling will have an impact on the epidemiological dynamics, this will consequently drive changes in the evolutionary dynamics. This evolutionary change may in turn impact the success of the management strategy. Moreover, the evolutionary changes associated with culling, if they impact virulence and transmission, may not only affect the focal species but could also lead to an increased impact from infection spillover into other populations, including farmed systems (Power & Mithcell, 2004; Woolhouse et al., 2001). Therefore, to provide insights into disease management and control (Galvani, 2003; Saad‐Roy et al., 2020), it is important to consider the role of culling on the evolution of virulence and transmission.

Previous studies have considered the evolutionary impact of indiscriminate culling on virulence and transmission (Bolzoni & De Leo, 2013; Rozins & Day, 2017; Shim & Galvani, 2009). In a single parasite model indiscriminate culling acts to reduce the average host lifespan and therefore, rather intuitively, selects for increased parasite transmission and virulence. This has been shown for general wildlife frameworks (Gandon et al., 2001) and for specific wildlife and livestock systems that consider avian influenza (Shim & Galvani, 2009) or Marek's disease virus (Rozins & Day, 2017). However, when there are high rates of superinfection, culling can lead to a decrease in evolved virulence (Bolzoni & De Leo, 2013). Studies exploring the evolutionary impact of culling specific epidemiological host classes are limited. Whilst in practice differentiating between infected and susceptible individuals may be difficult, or inefficient, in some systems culling can be targeted and this could lead to differences between the evolutionary outcomes. Furthermore, theory on the evolution of virulence typically focuses on wildlife systems (Cressler et al., 2016), where the host is typically regulated by the parasite and other intrinsic density‐dependent processes. In livestock systems, the population density may be externally maintained at constant levels, as broadly, the loss of individuals through natural death or disease‐induced mortality is balanced by restocking. There has been relatively little examination of how these different population processes impact the evolution of virulence although Mennerat et al. (2017) showed that in a farmed salmon population, parasites may evolve to a higher infestation and virulence level than they would in the wild.

We aim to compare the evolutionary consequences of targeted culling/harvesting in wildlife and livestock systems with endemic infection. Densities in the general wildlife system will be regulated by the parasite, whilst in the livestock system they will be maintained at a constant level, with losses through natural or disease‐induced mortality compensated by restocking. We consider models with different transmission routes, including density‐dependent, frequency‐dependent, and vertical transmission, as many parasites can transmit through mixed modes of transmission (Ebert, 2013). A key issue in livestock systems is the importation and restocking of infected individuals (UK Government, 2015) which acts in a similar manner to vertical transmission. Studies have shown that vertical transmission can select for lower virulence (Agnew & Koella, 1997; Ebert, 2013; Pagán et al., 2014; Stewart et al., 2005) and therefore we will investigate how this interacts with the evolutionary pressure from culling. We compare indiscriminate culling and the targeted culling of specific infection classes of the host and address the question of how parasite evolution may affect the level of culling required to eradicate an infectious disease. We aim to provide a comprehensive characterization of the role culling has on the evolution of parasite transmission and virulence, and its impact on the management of infectious disease in wildlife and livestock systems.

## 2 WILDLIFE AND LIVESTOCK MODEL

We detail a model framework that represents a general wildlife (Equation 1) and livestock (Equation 2) epidemiological system. We assume that the populations can be categorized based on their infection status, with S representing individuals who are uninfected but susceptible to infection, I, infected individuals who are infectious and R, individuals who have recovered from the infection and have acquired immunity. The wildlife epidemiological system is as follows:
(1)

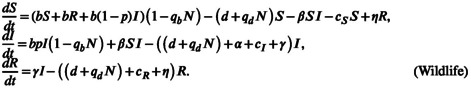

Here, N=S+I+R denotes the total population density, b, the maximum birth rate and d, the constant death rate. Population growth can be regulated through a density‐dependent term on birth, $q_{b}$, or death, $q_{d}$.

For the infection process, we assume susceptible individuals can progress to an infected class through contact with infected individuals with coefficient β. Here we assume density‐dependent infection transmission, but later we also consider frequency‐dependent transmission (see Supplementary material Section S.6). We also include vertical transmission of infection, where a proportion, p, of births from infected individuals are born infected. Infected individuals can suffer disease‐induced mortality, at rate α, or recover from the infection and progress to a recovered and immune class, at rate γ. Immunity to infection wanes over time, with individuals progressing back to a susceptible class at rate η.

We assume culling occurs at rates, $c_{S},c_{I}$ and $c_{R}$ for the susceptible, infected and recovered population classes, respectively. We can model the effect of indiscriminate culling or targeted culling of specific classes, such as a test and remove culling strategy based on infectious or antibody status that has been applied in wildlife and livestock systems (Che'Amat et al., 2016; Lambert et al., 2021; Miguel et al., 2020).

We represent the livestock population dynamics and the role of culling in a simplified, general, manner that captures the property of maintaining a constant population size through restocking to compensate for losses due to natural death, disease‐induced mortality or culling. Livestock populations show individual and herd resilience to infection that can be modelled as an SIR framework (Doeschl‐Wilson et al., 2021) and can represent pathogens that lead to immunity (for example, tick‐borne disease (Bram, 1983) and Rift Valley fever virus (Bett et al., 2017)). The livestock epidemiological system is as follows:
(2)

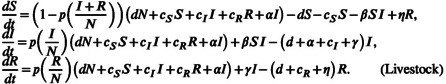

Here, N=S+I+R represents the total population density, but unlike the wildlife model this is assumed to be constant since the population is restocked to maintain the same total density. The rate of restocking matches the rate of death through natural causes, d, disease‐induced mortality, α, and culling, $c_{S},c_{I},c_{R}$. Infection transmission, recovery and waning immunity are all as detailed in the wildlife model. As our main emphasis is to investigate the evolution of virulence in response to different culling strategies, we assume culling to be a continuous process. While culling rates may vary at different times of the year, it can be considered an ongoing process and integral parts of herd management (Wakchaure et al., 2015). However, we recognize that culling procedures can be more complex than the continuous approach assumed in this study and may include pulse or seasonal culling events. The continuous culling procedure we employ provides a straightforward way to generate baseline theory on the implications of culling on pathogen evolution. We assume that restocking may include individuals that either are or have previously been infected, controlled by the parameter p. When p=0 all restocked individuals are susceptible. When p=1 restocking occurs to all classes relative to their density. Therefore, when p>0 restocking acts in a similar manner to vertical transmission. Note, to conduct a model analysis using the methods of adaptive dynamics we assume that restocked individuals are infected with the same strain of infection as our focal population (we conjecture that this will only change the rate of progression towards the evolutionary singular value and so not affect the results in our study).

To increase the generality of our results we undertake a sensitivity analysis for different rates of recovery to immunity and rates of loss of immunity. This can capture a wide range of infection processes (SI, SIR, SIRS) for different parameter combinations. We choose a general parameter set that ensures the infection persists at an endemic level in both model frameworks. We choose a low and high value for the rate of recovery from infection, γ, to represent a long and short infection period, respectively. We also choose the rate of waning immunity, η, to be zero or positive, to represent lifelong immunity or waning immunity, respectively. We allow restocking to include infected and recovered individuals, as the movements of live animals between farms is known to be one of the main routes of infection transmission (Bartlett et al., 2022; Fèvre et al., 2006) and is a major concern at local, national and global level (Huber et al., 2022). We then vary the rates of culling and assess the evolution of infection transmission and virulence for different parameter combinations. The parameters are detailed in Table 1. Our parameters are general, and not representative of a specific system. A strength of general theoretical studies is that the findings can be interpreted and discussed in terms of the biological processes that drive the model results and this makes the general findings applicable to a wide range of systems.

**TABLE 1:** Parameter definitions and default values for the wildlife infection model and livestock infection model (see Equations 1 and 2).

Parameter	Description
*b* = 10	Maximum birth rate
*d* = 1	Death rate
*K* = 100	Carrying capacity in the absence of infection and culling (wildlife model)
*N* = 100	Total constant population density (livestock model)
*q_b_* = 0.009	Susceptibility to crowding
*q_d_* = 0	Strength of density‐dependent death
$c_{S},c_{I},c_{R}$	Culling rate of susceptible, infected and recovered individuals
*p*	Proportion of vertical transmission (wildlife)/coefficient of restocking of *I* and *R* (livestock)
γ = 1 or 5	Rate of recovery
η = 0 or 5	Rate of waning immunity
β	Infection transmission coefficient (see Equation 3)
α	Virulence

**TABLE 2:** Parameter definitions and default values for the trade‐off function represented by Equation (3).

Parameter	Value	Description
a	−0.5	Strength of curvature for the trade‐off function
$\beta_{\text{min}}$	0.1	Minimum value for transmission coefficient, β
$\beta_{\text{max}}$	0.5	Maximum value for transmission coefficient, β
$\alpha_{\text{min}}$	0	Minimum value for virulence, α
$\alpha_{\text{max}}$	10	Maximum value for virulence, α

### 2.1 Evolutionary trade‐off function

**FIGURE 1:**
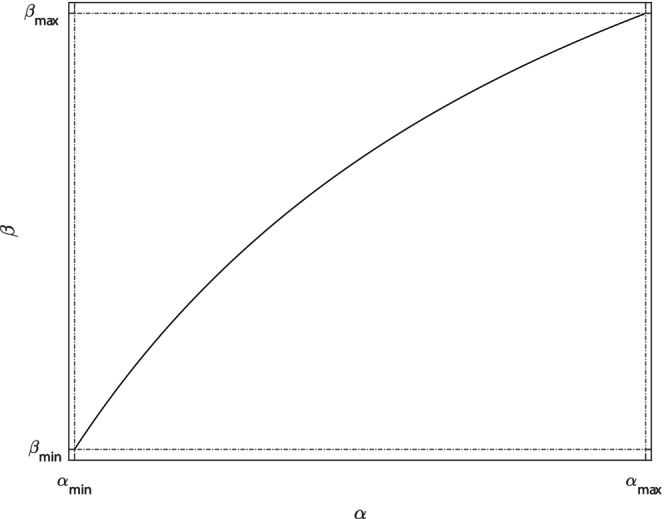
The trade‐off function, Equation (3), between virulence, α and infection transmission, β. Parameter are given in Table 2.

To explore the evolution of virulence, we include an evolutionary trade‐off between the disease‐induced mortality rate, α and the transmission coefficient β (Anderson & May, 1982; Cressler et al., 2016). We choose a generic trade‐off function that follows the same methodology of that outlined in White et al. (2006) (see Equation 3 and Figure 1). We chose a value for the curvature parameter, a=‐0.5  in Equation (3), that ensures the trade‐off has accelerating costs. The trade‐off function is as follows:

(3) $$\beta =f\left(\alpha \right)=\beta_{\min}+\frac{\left(\beta_{\max}-\beta_{\min}\right)\left(1-\frac{\alpha_{\max}-\alpha }{\alpha_{\max}-\alpha_{\min}}\right)}{1+a\frac{\alpha_{\max}-\alpha }{\alpha_{\max}-\alpha_{\min}}}.$$

### 2.2 Evolutionary dynamics

Our aim is to explore the effect of culling on the evolution of infection, using the trade‐off function detailed in Equation (3) and applying the methods of adaptive dynamics (Geritz et al., 1998; Metz et al., 1996). Adaptive dynamics assumes a separation of the epidemiological and evolutionary time scales and that a mutant strain, with small phenotypic variation from the resident strain, is rare and attempts to invade the resident system at its dynamical attractor (a stable steady state in this study) (Geritz et al., 1998; Metz et al., 1996). To understand how the infection will evolve we need to derive the fitness function of a mutant strain of the infection and determine the conditions when the mutant can invade the resident population. We assume the mutant parameters are $\bar{\beta }$, $\bar{\alpha }$ and the resident parameters are β, α. For the wildlife model framework, the mutant fitness function, $\bar{s}$, can be derived and is given by:

(4) $$\bar{s}=\mathrm{bp}\left(1-q_{b}N\right)+\bar{\beta }S-\left(\left(d+q_{d}N\right)+\bar{\alpha }+c_{I}+\gamma \right),$$

where S denotes the steady state density of the resident susceptible population. Given that $\beta =f\left(\alpha \right)$, from our trade‐off function, we can determine the local directional gradient of the mutant fitness:

$$\frac{\partial \bar{s}}{\partial \bar{\alpha }}=f^{\prime }\left(\bar{\alpha }\right)S-1.$$

This will reach a singular strategy, $\alpha^{*}$, when the following condition is met:

(5) $$f^{\prime }\left(\alpha^{*}\right)=1/S^{*},$$

where $S^{*}$ denotes the steady state value of the susceptible population evaluated at the singular strategy, $\alpha^{*}$.

We can undertake a similar analysis for the livestock model and the condition for the singular strategy is again represented by Equation (5). Note however, that the definition and value of $S^{*}$ will be different in the wildlife and livestock model frameworks due to the difference in regulation between each framework. For further details on the evolutionary analysis, see Sections S1.1 and S1.2.

For $\alpha^{*}$ to be an evolutionary attractor we need to ensure that it is evolutionary and convergence stable (Geritz et al., 1998). These stability conditions are shown in the supplementary material (see S1.2). In the analysis that follows, we make the following assumptions. That the trade‐off function is chosen such that the singular strategy, in both the wildlife and livestock model frameworks, is evolutionary and convergence stable (Bowers et al., 2005) and that all parameter combinations on the trade‐off support a stable, endemic, population steady state. When these assumptions are satisfied our results are qualitatively similar for a wide range of trade‐off curves.

For the model results, we use the computer program MATLAB 2022a to solve Equation (5) for the singular level of virulence (which requires that we also solve the system of Equations (1) or (2) for the steady state value of $S^{*}$). We also use these methods to calculate $R_{0}$ (see Equations S10 or S11) and the total population density for different parameter combinations.

## 3 IMPACT OF VERTICAL TRANSMISSION ON THE EVOLUTION OF VIRULENCE

Previous model studies that represent wildlife systems have shown that the inclusion of vertical transmission can reduce the evolutionarily stable level of virulence (Agnew & Koella, 1997; Ebert, 2013; Pagán et al., 2014; Stewart et al., 2005). The livestock equivalent for vertical transmission is restocking, but this can introduce infected or recovered individuals, that reflect the endemic nature of infection in the wider population.

**FIGURE 2:**
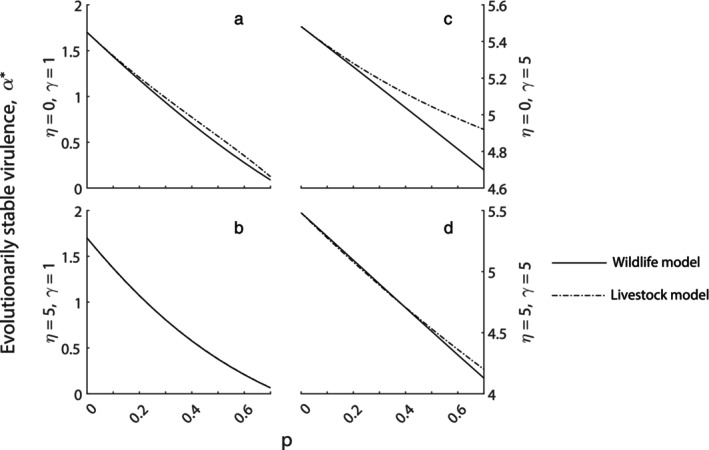
Evolved level of virulence, $\alpha^{*}$, for a varying level of vertical transmission, p, under a wildlife model with a density‐dependent birth rate (solid line) or for a varying level of restocking of infected and recovered individuals, p, in a livestock model (dot‐dashed line) (see Equations 1 and 2 respectively). Results are shown for different infection types with (a) η=0, γ=1, (b) η=5, γ=1, (c) η=0, γ=5 and (d) η=5, γ=5. When not varied in the figure, parameters are taken from Tables 1 and 2, with β given by the trade‐off function, Equation (3).

For the wildlife model framework with density‐dependent birth, increasing the level of vertical transmission reduces the evolutionarily stable level of virulence (see Figure 2). Here, the addition of vertical transmission reduces the reliance on direct transmission and so the parasite evolves to reduce direct transmission, β, and therefore virulence, α. For the wildlife model with density‐dependent death the results are similar in that the evolved level of virulence decreases as vertical transmission increases (see Section S.2). We note that the magnitude of the decrease in virulence for the model with density‐dependent death is reduced compared to the model with density‐dependent birth, likely due to the lack of compensatory growth due to culling in the absence of density‐dependent birth (Tanner et al., 2019).

For the livestock model framework with waning immunity, increasing the level of restocking to the I and R class acts to reduce virulence in a similar manner to increasing the level of vertical transmission in the wildlife model (Figure 2). When there is lifelong immunity and a short infection period the reduction in evolved virulence as restocking of I and R increases is less pronounced (Figure 2c). Here, at the endemic steady state the population contains a high proportion of immune (R) individuals and therefore restocking favors immune over infected individuals as p increases and negates some of the benefit to the pathogen of restocking infected individuals.

## 4 IMPACT OF CULLING ON THE EVOLUTION OF VIRULENCE

To examine the impact of culling on the evolution of infection transmission and virulence we consider four different culling scenarios: (i) culling the susceptible population only, (ii) culling the infected population only, (iii) culling the recovered population only and (iv) culling all classes at the same rate (indiscriminate culling). For targeted culling, the respective culling rate is set to a constant c. For indiscriminate culling, we set $c_{S}=c_{I}=c_{R}=c/3$ such that the total culling effort, $c_{S}+c_{I}+c_{R}$, is the same for targeted and indiscriminate culling. We explore the effect culling has on the evolution of virulence under a wildlife and livestock model framework.

**FIGURE 3:**
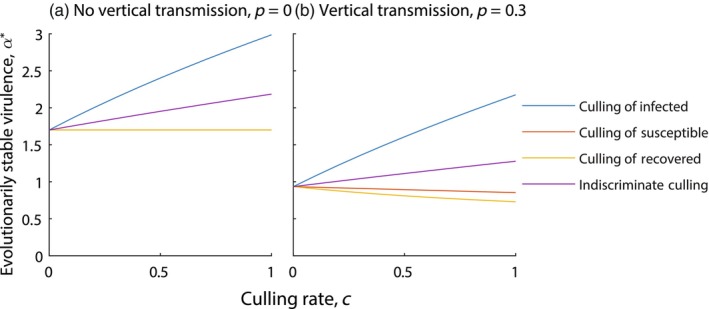
Evolved level of virulence, $\alpha^{*}$, for varying rates of culling, under a wildlife model framework (see Equation 1). Results are shown in (a) the absence of vertical transmission and (b) with vertical transmission, p=0.3. Different types of culling are indicated with (blue) culling of infected individuals only, (red) culling of susceptible individuals only, (yellow) culling of recovered individuals only and (purple) indiscriminate culling. When not varied in the figure, parameters are taken from Tables 1 and 2, with γ=1 and η=0.

For the wildlife model framework, in the absence of vertical transmission, culling susceptible or recovered individuals has no effect on the evolution of virulence (see Figure 3a). For this model set‐up the steady state density of the susceptible population, $S^{*}$, is defined as follows:

(6) $$S^{*}=\frac{d+\alpha^{*}+c_{I}+\gamma }{\beta^{*}}\;\;\;\;\;\;\;\;\;\;\;\;\;\;\;\;\;\;\;\;\;\left(p=q_{d}=0\right)$$

and is independent of $c_{S}$ and $c_{R}$. Therefore, the evolutionary fixed point determined from expression (5) is also independent of $c_{S}$ and $c_{R}$. However, culling infected individuals does change $S^{*}$, and this leads to the evolution of higher virulence (and higher infection transmission) as $c_{I}$ increases. For indiscriminate culling, the culling rate of infected individuals is reduced (compared to targeted culling of infected individuals only) and so the increase in virulence is less pronounced as the level of indiscriminate culling increases.

Similar dynamics are seen when vertical transmission is included for culling of infected individuals (see Figure 3b). However, culling of susceptible or recovered individuals now reduces the evolved level of virulence since culling leads to a reduction in the steady state density of susceptibles, $S^{*}$. Indiscriminate culling is a combination of results from culling the different classes. As such, the increase in virulence associated with culling infected individuals is tempered by the decrease associated with the culling of susceptible and recovered hosts.

**FIGURE 4:**
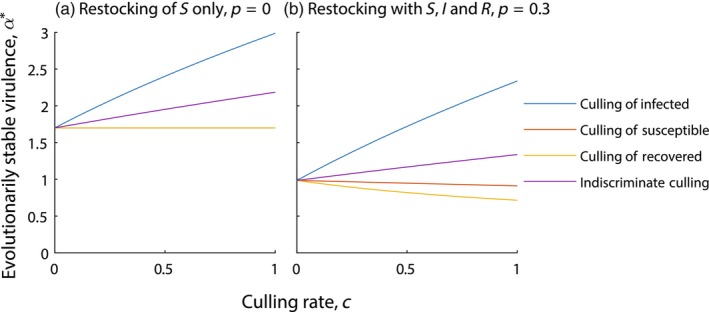
Evolved level of virulence, $\alpha^{*}$, for varying rates of culling, under a livestock model framework (see Equation 2). Results are shown when (a) restocking with S only, p=0 and (b) restocking with S, I and R, p=0.3. Different types of culling are indicated with (blue) culling of infected individuals only, (red) culling of susceptible individuals only, (yellow) culling of recovered individuals only and (purple) indiscriminate culling. When not varied in the figure, parameters are taken from Tables 1 and 2, with γ=1 and η=0.

The impact of culling in the livestock model has similar effects to the wildlife model (compare Figures 3a and 4a and Figures 3b and 4b). We see an increase in evolved virulence when infected individuals are culled and either no impact (when p=0) or a decrease in evolved virulence when susceptible and recovered individuals are culled when restocking can include the I and R classes (p>0). Here, culling susceptible and recovered individuals leads to an increase in the restocking rate of infected individuals and so the parasite can evolve to a lower level of transmission and virulence. Furthermore, culling recovered individuals also leads to an increase in the susceptible density, $S^{*}$, which selects for a reduction in direct transmission and virulence and explains why the reduction in virulence is more pronounced when culling recovered compared to susceptible individuals. These evolutionary dynamics are consistent for a range of parameter values (see Section S.3).

Other general properties under both model frameworks (see Section S.3) are that increasing the rate of recovery results in an increased evolved level of virulence (and transmission) as the parasite requires higher transmission to counter the decreased duration in the infected class. Increasing the rate of waning immunity (η) has no impact on the evolved level of virulence when p=0 since it does not affect the susceptible steady state density. When p>0 increases in the rate of waning immunity reduces the magnitude (but not the trends) of change in the level of evolved virulence as the culling rate increases. Note, a decrease in the total underlying density, by decreasing the carrying capacity in the wildlife model or decreasing the total density in the livestock model, has no qualitative impacts and minimal quantitative differences (results not shown).

## 5 IMPACT OF CULLING AND EVOLUTION ON WILDLIFE AND LIVESTOCK MANAGEMENT PRACTICES

Culling can be used for management practices in many ecological and epidemiological scenarios. From an epidemiological perspective culling can be used to eradicate infection by reducing the number of contacts an infected individual can make (Anderson et al., 1981; Lloyd‐Smith et al., 2005; McCallum, 2016). From an ecological perspective it can reduce the amount of over‐abundant ‘pest’ species or can be used to harvest a species (Caughley & Sinclair, 1994; Fryxell et al., 2014). However, culling a population where infection is present can cause the infection to evolve, potentially making it harder to achieve the management objectives. Here, we explore the effects of evolution that arise due to culling and the consequences it has on livestock and wildlife management.

**FIGURE 5:**
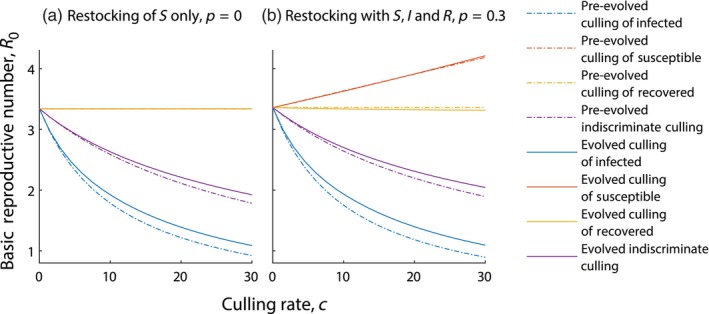
The basic reproductive number, $R_{0}$, for varying rates of culling, under a livestock model framework (see Equation 2). Results are shown when (a) restocking with S only, p=0 and (b) restocking with S, I and R, p=0.3. Different types of culling are indicated with (blue) culling of infected individuals only, (red) culling of susceptible individuals only, (yellow) culling of recovered individuals only and (purple) indiscriminate culling. We show results when virulence evolves to the evolutionary singular strategy in response to culling (solid line) and when virulence is fixed at the evolutionary singular strategy in the absence of culling (dot‐dashed line). When not varied in the figure, parameters are taken from Tables 1 and 2, with γ=5 and η=5.

For livestock populations, the aim of management strategies is to eradicate infection. For this we need to reduce the basic reproductive number, $R_{0}$, of the infection to below unity (the derivation of $R_{0}$ is detailed in Section S.4). Culling infected individuals has the greatest impact on reducing the reproductive number (see Figure 5). Culling susceptible or recovered individuals only has a limited effect, highlighting the importance of identifying the infection status of an individual before culling. Note, however, that culling infected individuals leads to parasite evolution that increases $R_{0}$. As such, evolution will make it harder to eradicate infection through culling. To successfully eradicate the infection, culling would need to be swift to limit the opportunity of parasite evolution. When we include restocking of infected or recovered individuals, culling susceptible individuals can lead to an increase in $R_{0}$. Here, the increased mortality of susceptible individuals leads to increased restocking of infected individuals which increases $R_{0}$.

For wildlife populations, if the aim is to reduce the environmental impact of an over‐abundant host, then the best culling strategy would be to target the population class that has the greatest impact on reducing the total population. If the aim is to harvest the population, then the opposite holds. The optimal culling strategy can depend on the way culling is recorded, where culling can be based on effort (the culling rate) or on the density of culling individuals (the culling rate multiplied by the density of the relevant class). We examine the impact of culling on population density in terms of the density culled per unit time, since when culling is measured by rate it is highly dependent on the density of the population classes.

**FIGURE 6:**
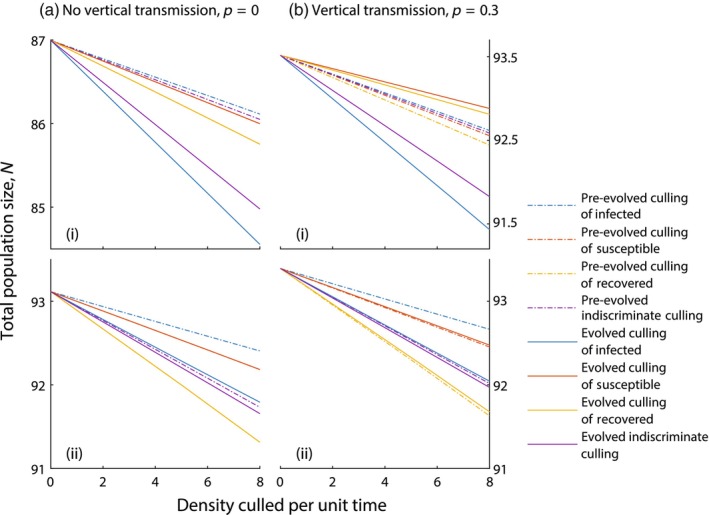
The total population density, N, for various densities culled per unit time, under a wildlife model framework (see Equation 1). Results are shown in (a) the absence of vertical transmission, p=0 and (b) with vertical transmission, p=0.3 and for different infection types with (i) η=5,γ=1 and (ii) η=0,γ=5. Different types of culling are indicated with (blue) culling of infected individuals only, (red) culling of susceptible individuals only, (yellow) culling of recovered individuals only and (purple) indiscriminate culling. We show results when virulence evolves to the evolutionary singular strategy in response to culling (solid line) and when virulence is fixed at the evolutionary singular strategy in the absence of culling (dot‐dashed line). When not varied in the figure, parameters are taken from Tables 1 and 2. Note, in the absence of vertical transmission, p=0, when culling either susceptible or recovered individuals, the lines representing the evolved and pre‐evolved states are the same.

In the absence of evolutionary effects, culling infected individuals has the least impact on reducing the total population size and culling recovered individuals has the greatest impact (see Figure 6). Here, culling the infected class removes the class with the highest overall death rate and reduces the force of infection and as such, reduces the impact of disease‐induced mortality at the population level. Culling recovered individuals removes individuals that were immune to infection and so has the greatest impact on reducing the total population size. When culling the infected class, evolution acts to increase virulence, leading to an increase in population level mortality and further population reductions. This increases the total mortality in the system and increases the population reduction. In the absence of vertical transmission, the culling of susceptible or recovered individuals does not lead to a change of virulence. However, with vertical transmission, evolution acts to decrease virulence, decreasing the population level mortality and mitigating some of the density reduction due to culling.

Note, results for the impact of culling on outcome management practice are shown for a wider range of parameters in Section S.5. Furthermore, all our results hold if we assume frequency‐dependent infection transmission in the wildlife model, instead of density‐dependent transmission (see Section S.6).

## 6 DISCUSSION

Given their capacity for rapid evolutionary change, a better understanding of the impacts of human interventions on the evolution of infectious disease is needed if we are to develop optimal management strategies. We have examined the effects of targeted culling/harvesting and cross generational transmission on the evolution of parasite virulence and transmission in wildlife hosts, that are regulated by the parasite, and livestock hosts, that are maintained at a constant population size through restocking. We developed general SIR and SIRS wildlife and livestock frameworks and have explored the evolution of parasite virulence through a classic virulence‐transmission trade‐off. Our key result is that targeted culling has contrasting impacts on the evolution of virulence and can therefore both help and hinder management strategies to control parasites or pest species or harvest populations. We also show that the reduction in the level of evolved virulence due to vertical transmission has clear parallels in livestock systems where restocking that includes infected individuals can lead to a decrease in the evolved level of virulence.

The effect of indiscriminate culling leading to the evolution of higher virulence has been well studied (Bolzoni & De Leo, 2013; Rozins & Day, 2017; Shim & Galvani, 2009) and our results confirm this finding holds in livestock as well as wildlife systems. This can be understood intuitively since indiscriminate culling effectively increases background mortality, shortening the infectious period and selecting for acute, highly transmissible, parasites (Cressler et al., 2016). However, we further show in our general model framework that the outcome is much more nuanced if culling is targeted on the specific susceptible, infected or recovered classes when there is vertical transmission or restocking that includes infected individuals. Culling infected individuals has a stronger effect than indiscriminate culling and also always selects for higher virulence in both wildlife and livestock system (Rozins & Day, 2017; Shim & Galvani, 2009). However, for both the wildlife and livestock models, targeted culling of the recovered or susceptible class can instead select for a reduced virulence when there is vertical transmission or restocking that includes infected individuals. This is a key result since it shows how different types of culling may have different evolutionary outcomes. It also has important management implications, as we typically want to avoid the selection of higher virulence when culling or harvesting. Our model considers culling that is independent of pathogen virulence. Wargo et al. (2021) showed that selection for low virulence could occur if culling is triggered when population mortality exceed a threshold. Here, there is preferential culling of hosts infected with highly virulent strains, leading to pathogen extinction, whereas hosts infected with low virulent strains avoid culling and the pathogen can persist.

Culling can be used as a management strategy to eradicate a parasite, particularly in livestock populations. Culling of susceptible or recovered individuals does little to eradicate the parasite and may aid its persistence, whereas culling infected individuals can reduce the reproductive number leading to parasite eradication. However, our results show that culling infected individuals selects for increased virulence and transmission, which in turn requires a stronger culling effort to eradicate the parasite. The implication of this is that evolution in response to culling makes the eradication of infection more difficult. This is a good example where neglecting evolutionary outcomes that arise through management interventions may be problematic. Clearly if a culling program is fast, there may not be time for mutations to occur. However, there is often likely to be standing genetic variation in parasite populations and so it is potentially dangerous to ignore the evolutionary outcomes in management strategies.

When the goal of culling is to reduce the abundance of a wildlife species (Caughley & Sinclair, 1994; Fryxell et al., 2014) targeting recovered individuals leads to the biggest reduction in total population density in the short‐term (before evolution has occurred). Culling the recovered class leaves the population more vulnerable to infection, which acts with culling to reduce the population size. However, culling recovered individuals leads to the evolution of reduced virulence and this counters the population reduction due to culling. In contrast, the culling of infected individuals leads to the smallest population reduction before any evolutionary processes have occurred. However, culling infected individuals leads to an increase in virulence and can increase population level mortality potentially leading to the greatest drop in population density. This is an example where evolution can promote the goals of an intervention strategy. For sustainable harvesting of a wildlife species, the goal is to limit the reduction in population density while taking a sustainable harvest (Caughley & Sinclair, 1994; Fryxell et al., 2014). This mirrors the goal of reducing the abundance of a wildlife species, and so targeting infected individuals will have the greatest success in the short‐term. In the absence of evolutionary effects, targeting infected individuals has the least effect on population density. However, culling infected individuals selects for increased virulence and an undesirable population reduction. Targeted culling of susceptible or recovered individuals can lead to a reduction in virulence and a reduced impact of culling on the population. This provides a second example of how evolution can promote the goals of an intervention strategy.

Our results confirm the well‐known finding for vertical transmission in a wildlife setting, that increased vertical transmission selects for lower virulence in the parasite (Agnew & Koella, 1997; Ebert, 2013; Pagán et al., 2014; Stewart et al., 2005). A further key finding is that we show that restocking, that includes infected individuals selects for a reduction in parasite virulence in livestock systems. Thus restocking in livestock systems acts in a similar manner to vertically transmitted infection in wildlife in that it increases the supply of infected individuals and therefore the parasite selects for a reduced level of direct transmission and virulence. However, selection for lower virulence and transmission in livestock populations for parasites that convey long‐term immunity is less marked since restocking can also enhance the density of the immune class.

In this paper, we have highlighted the importance of virulence evolution on infectious disease epidemiology and control. Culling based on the infection status should be a critical consideration when developing infectious disease control strategies. Evolutionary outcomes are nuanced and can either hinder or enhance the desired management outcome and our work emphasizes that we should build new theory to examine the impacts of the wide range of different population processes that we see in managed and natural populations. Our work has examined two simple situations, but there is much more to be done to produce a comprehensive theory of the evolution of virulence in managed systems under different disease management intervention strategies.

## CONFLICT OF INTEREST STATEMENT

The authors declare no conflict of interest.

## Supporting information


Data S1.

## Data Availability

No data was required for this study.
