# Supplementary material for: The evolution of parasite virulence under targeted culling and harvesting in wildlife and livestock
Source: Evol Appl. 2023 Sep 28;16(10):1697–707. doi: 10.1111/eva.13594 (PMC10660816; doi:10.1111/eva.13594)
Supplement: Supplementary file 1 — Data S1. [file EVA-16-1697-s001.pdf]

Supplementary Material: The evolution of parasite virulence under culling and harvesting in wildlife and livestock

S.1 Wildlife and livestock models: evolutionary analysis

S.1.1 Fitness expression

The epidemiological wildlife model, including the dynamics of a resident strain, I (with parameters β and α) and the mutant infection strain, $\bar{I}$ (with parameters $\bar{\beta}$ and $\bar{\alpha}$), is shown as follows:

$$\begin{aligned}
\frac{dS}{dt} &= (bS + bR + b(1-p)(I+\bar{I}))(1-q_bN) - (d+q_dN)S - (\beta I + \bar{\beta}\bar{I})S - c_S S + \eta R,\\
\frac{dI}{dt} &= bpI(1-q_bN) + \beta SI - ((d+q_dN) + \alpha + c_I + \gamma)I,\\
\frac{dR}{dt} &= \gamma(I+\bar{I}) - ((d+q_dN) + c_R + \eta)R,\\
\frac{d\bar{I}}{dt} &= bp\bar{I}(1-q_bN) + \bar{\beta}S\bar{I} - ((d+q_dN) + \bar{\alpha} + c_I + \gamma)\bar{I}.
\end{aligned} \tag{S.1}$$

From this we can determine the fitness expression for the mutant infection strain by finding conditions for the instability of the resident strain steady state in the absence of a mutant population, $(S, I, R, \bar{I} = 0)$. The stability matrix can be represented by:

$$J(S,I,R,0) = \left.\begin{pmatrix}
\frac{\partial \dot{S}}{\partial S} & \frac{\partial \dot{S}}{\partial I} & \frac{\partial \dot{S}}{\partial R} & \frac{\partial \dot{S}}{\partial \bar{I}}\\
\frac{\partial \dot{I}}{\partial S} & \frac{\partial \dot{I}}{\partial I} & \frac{\partial \dot{I}}{\partial R} & \frac{\partial \dot{I}}{\partial \bar{I}}\\
\frac{\partial \dot{R}}{\partial S} & \frac{\partial \dot{R}}{\partial I} & \frac{\partial \dot{R}}{\partial R} & \frac{\partial \dot{R}}{\partial \bar{I}}\\
\frac{\partial \dot{\bar{I}}}{\partial S} & \frac{\partial \dot{\bar{I}}}{\partial I} & \frac{\partial \dot{\bar{I}}}{\partial R} & \frac{\partial \dot{\bar{I}}}{\partial \bar{I}}
\end{pmatrix}\right|_{(S,I,R,0)} = \left.\begin{pmatrix}
\frac{\partial \dot{S}}{\partial S} & \frac{\partial \dot{S}}{\partial I} & \frac{\partial \dot{S}}{\partial R} & \frac{\partial \dot{S}}{\partial \bar{I}}\\
\frac{\partial \dot{I}}{\partial S} & \frac{\partial \dot{I}}{\partial I} & \frac{\partial \dot{I}}{\partial R} & \frac{\partial \dot{I}}{\partial \bar{I}}\\
\frac{\partial \dot{R}}{\partial S} & \frac{\partial \dot{R}}{\partial I} & \frac{\partial \dot{R}}{\partial R} & \frac{\partial \dot{R}}{\partial \bar{I}}\\
0 & 0 & 0 & \frac{\partial \dot{\bar{I}}}{\partial \bar{I}}
\end{pmatrix}\right|_{(S,I,R,0)} \tag{S.2}$$

In all model results we have chosen parameters such that in the absence of the mutant strain, the resident population is at a stable, endemic, steady state. Therefore, the eigenvalues of the upper 3x3 part of this matrix are negative (albeit too complex to display here). The condition for the invasion of the resident strain by the mutant strain, in our model framework, therefore depends on the sign of the fourth eigenvalue, (expression S.3), which we can define as the fitness expression for the mutant infection strain, $\bar{s}$.

$$\bar{s} = \lambda_4 = \left.\frac{\partial \dot{\bar{I}}}{\partial \bar{I}}\right|_{(S,I,R,0)} = bp(1-q_bN) + \bar{\beta}S - ((d+q_dN) + \bar{\alpha} + c_I + \gamma). \tag{S.3}$$

The epidemiological livestock model, including the dynamics of a resident strain, I (with parameters β and α) and the mutant infection strain, $\bar{I}$ (with parameters $\bar{\beta}$ and $\bar{\alpha}$), is shown as follows:

$$\begin{aligned}
\frac{dS}{dt} &= \left(1 - p\left(\frac{I+\bar{I}+R}{N}\right)\right)\left(dN + c_S S + c_I(I+\bar{I}) + c_R R + \alpha I + \bar{\alpha}\bar{I}\right) - dS - c_S S - (\beta I + \bar{\beta}\bar{I})S + \eta R,\\
\frac{dI}{dt} &= p\left(\frac{I}{N}\right)\left(dN + c_S S + c_I(I+\bar{I}) + c_R R + \alpha I + \bar{\alpha}\bar{I}\right) + \beta SI - (d + \alpha + c_I + \gamma)I,\\
\frac{dR}{dt} &= p\left(\frac{R}{N}\right)\left(dN + c_S S + c_I(I+\bar{I}) + c_R R + \alpha I + \bar{\alpha}\bar{I}\right) + \gamma(I+\bar{I}) - (d + c_R + \eta)R,\\
\frac{d\bar{I}}{dt} &= p\left(\frac{\bar{I}}{N}\right)\left(dN + c_S S + c_I(I+\bar{I}) + c_R R + \alpha I + \bar{\alpha}\bar{I}\right) + \bar{\beta}S\bar{I} - (d + \bar{\alpha} + c_I + \gamma)\bar{I}.
\end{aligned} \tag{S.4}$$

Following the same procedure as for the wildlife fitness function we can determine the fitness function for a mutant strain in a livestock system to be:

$$\bar{s} = p\left(\frac{1}{N}\right)(dN + c_S S + c_I I + c_R R + \alpha I) + \bar{\beta}S - (d + \bar{\alpha} + c_I + \gamma). \tag{S.5}$$

S.1.2 Conditions for evolutionary attractor

In the main text we show that given the trade-off $\beta = f(\alpha)$ we can determine the local directional gradient of the mutant fitness:

$$\frac{\partial \bar{s}}{\partial \bar{\alpha}} = f'(\bar{\alpha})S - 1. \tag{S.6}$$

and therefore the singular strategy, α^*, occurs when the following condition is met.

$$f'(\alpha^*) = 1/S^*, \tag{S.7}$$

where S^* denotes the steady state value of the susceptible population evaluated at the singular strategy, α^*.

For an evolutionary attractor we require that the singular strategy, α^*, is both evolutionary stable (ES) and convergence stable (CS) [Bowers et al., 2005, Geritz et al., 1998, Metz et al., 1996]. We outline these conditions below, which are the same for both the wildlife and livestock model:

ES requires:

$$\left.\frac{\partial^2 \bar{s}}{\partial \bar{\alpha}^2}\right|_{\alpha=\bar{\alpha}=\alpha^*} = \left(f''(\bar{\alpha})S\right)|_{\alpha=\bar{\alpha}=\alpha^*} = f''(\alpha^*)S^* < 0. \tag{S.8}$$

CS requires:

$$\left.\left(\frac{\partial^2 \bar{s}}{\partial \alpha^2} - \frac{\partial^2 \bar{s}}{\partial \bar{\alpha}^2}\right)\right|_{\alpha=\bar{\alpha}=\alpha^*} = -\frac{2}{S^*}\left.\frac{\partial S}{\partial \alpha}\right|_{\alpha=\bar{\alpha}=\alpha^*} - 2f''(\alpha^*)S^* > 0. \tag{S.9}$$

We choose a trade-off to ensure that the singular strategy, in both the wildlife and livestock model frameworks, is evolutionary and convergence stable [Bowers et al., 2005].

S.2 Impact of vertical transmission on the evolution of virulence

In the main text we show the evolved level of virulence for increases in p for a livestock model and a wildlife model with density-dependent birth. In Figure S.1 we also show the change in the evolved level of virulence for a wildlife model with density-dependent death ($q_b = d = 0, q_d = b/K$). In the wildlife model with density-dependent death the birth rate, $b = 1$, is adjusted such that the total density in the absence of infection, $K = 100$, is consistent across the three models.

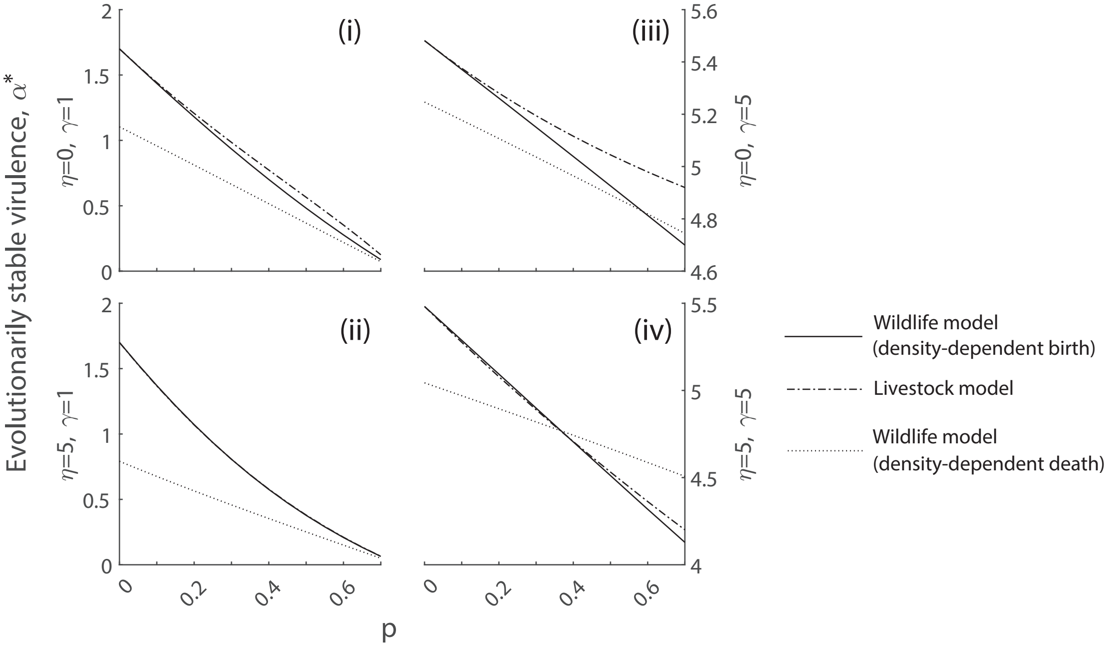

Figure S.1: Evolved level of virulence, α^*, for a varying level of vertical transmission, p, under a wildlife model with a density-dependent birth rate (solid line), a wildlife model with a density-dependent death rate (dashed line), or for a varying level of restocking of infected and recovered individuals, p, in a livestock model (dot-dashed line) (see equations 1 and 2, respectively). Results are shown for different infection types with (i) $\eta = 0, \gamma = 1$, (ii) $\eta = 5, \gamma = 1$, (iii) $\eta = 0, \gamma = 5$ and (iv) $\eta = 5, \gamma = 5$. When not varied in the figure, parameters are taken from Tables 1 and 2, with β given by the trade-off function, equation (3). In the wildlife model with density-dependent death the maximum birth rate takes the values $b = 1$.

S.3 Impact of culling on the evolution of virulence

In the main text we show results for the impact of culling on the evolution of virulence for the wildlife model (Figure 3) and the livestock model (Figure 4). Here we show the results for a wider set of parameters for the wildlife (see Figure S.2) and livestock (see Figure S.3) model frameworks.

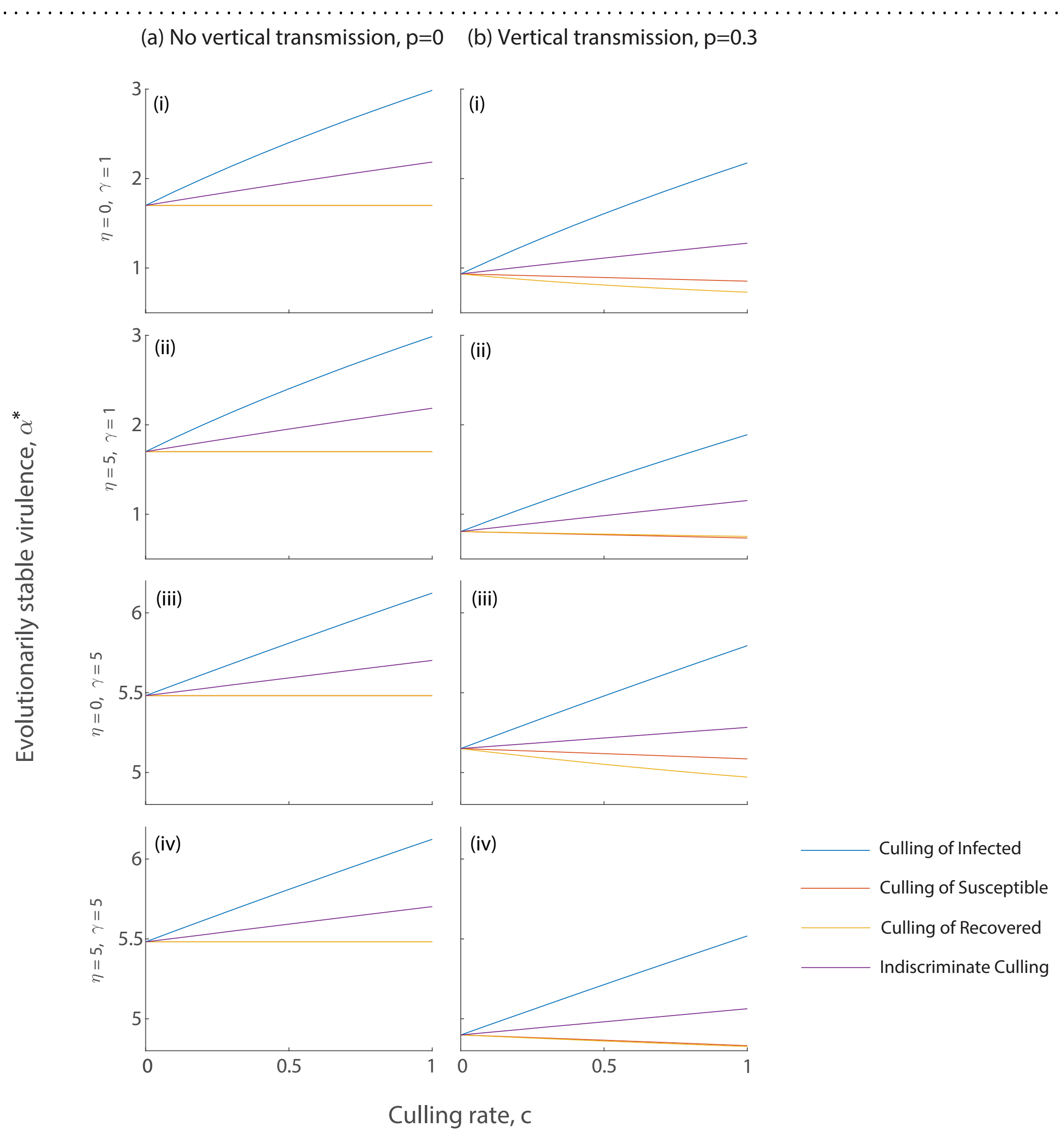

Figure S.2: Evolved level of virulence, α^*, for varying rates of culling, under a wildlife model framework with density-dependent transmission (see equations 1). Results are shown for (a) no vertical transmission, $p = 0$ and (b) with vertical transmission, $p = 0.3$. Different infection types are shown with (i) $\eta = 0, \gamma = 1$, (ii) $\eta = 5, \gamma = 1$, (iii) $\eta = 0, \gamma = 5$ and (iv) $\eta = 5, \gamma = 5$. Different types of culling are indicated with (blue) culling of infected individuals only, (red) culling of susceptible individuals only, (yellow) culling of recovered individuals only and (purple) indiscriminate culling. When not varied in the figure, parameters are taken from Tables 1 and 2 in the main text.

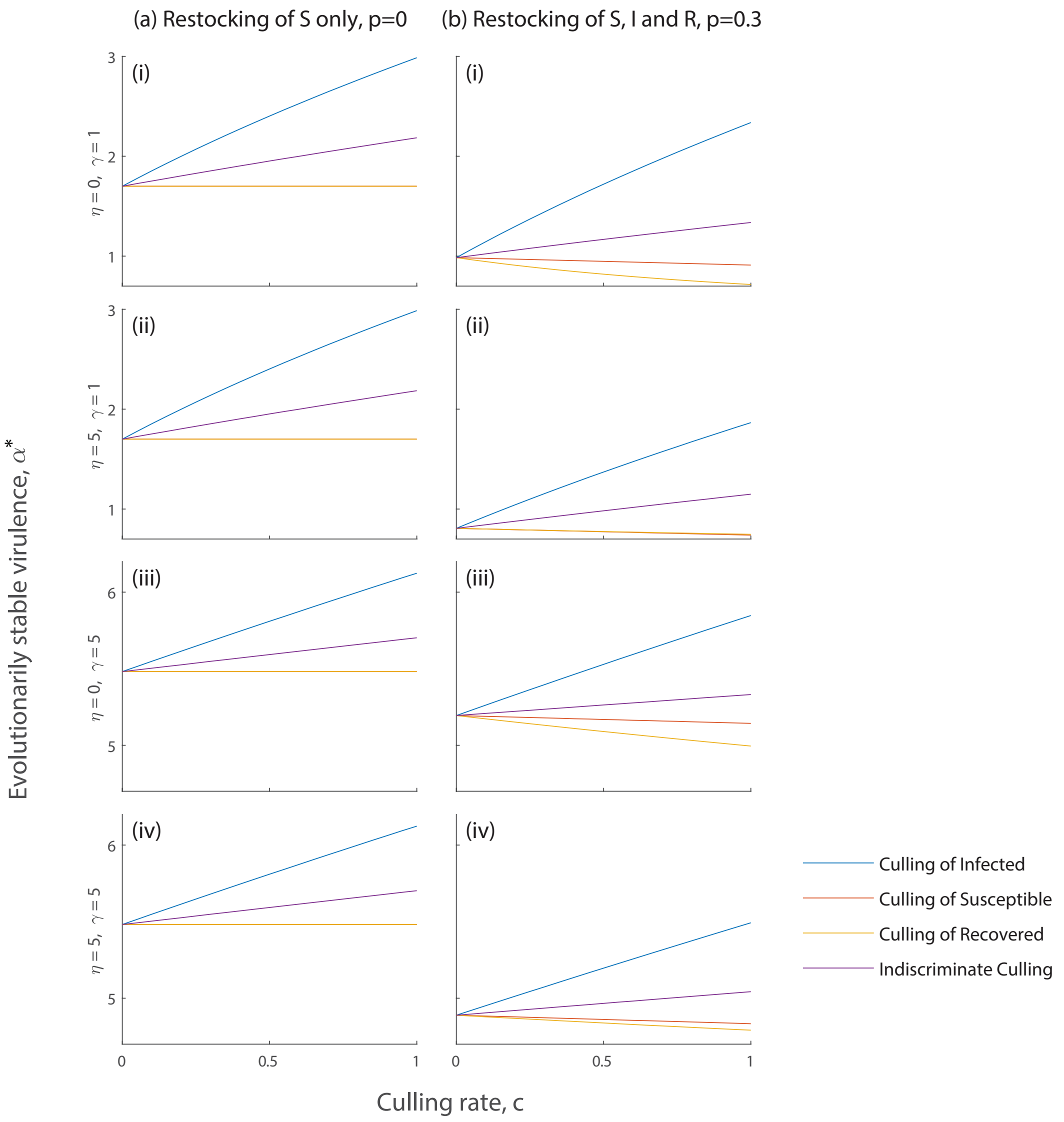

Figure S.3: Evolved level of virulence, α^*, for varying rates of culling, under a livestock model framework (see equations 2). Results are shown for (a) restocking with susceptible individuals (only) and (b) restocking that includes infected and recovered individuals, $p = 0.3$, and for different infection types with (i) $\eta = 0, \gamma = 1$, (ii) $\eta = 5, \gamma = 1$, (iii) $\eta = 0, \gamma = 5$ and (iv) $\eta = 5, \gamma = 5$. Different types of culling are indicated with (blue) culling of infected individuals only, (red) culling of susceptible individuals only, (yellow) culling of recovered individuals only and (purple) indiscriminate culling. When not varied in the figure, parameters are taken from Tables 1 and 2 in the main text.

S.4 Derivation of the basic reproductive number

We determine the basic reproductive number, R_0, for the wildlife model with density-dependent transmission (equations (1)) and the livestock model (equations (2)) as detailed in the main article:

Wildlife model

We can determine an explicit expression for the reproductive ratio, $\mathcal{R}_0$, using the next-generation matrix approach (NGM) [Diekmann et al., 2010], where $\mathcal{R}_0$ is given by the spectral radius of the matrix $\mathbf{FV^{-1}}$ evaluated at the disease-free steady state.

$$\begin{aligned}\mathcal{R}_0 &= \rho\left(\mathbf{FV^{-1}}(S^*,0,0)\right)\\ &= \frac{pb(d(q_b+q_d)+c_S q_b)+\beta(b-d-c_S)}{bq_b(d+\gamma+c_I+\alpha)+dq_d(b+\gamma+\alpha+c_I-c_S)},\end{aligned} \tag{S.10}$$

where

$$\mathbf{F} = \begin{bmatrix} pb(1-q_bN)-pbq_bI+\beta S & -pbq_bI \\ 0 & 0 \end{bmatrix},$$
$$\mathbf{V} = \begin{bmatrix} q_dI+d+q_dN+\alpha+c_I+\gamma & dq_dI \\ q_dR-\gamma & q_dR+d+q_dN+c_R+\eta \end{bmatrix}$$

and $S^* = \frac{b-c_S-d}{bq_b+dq_d}$ is the disease-free steady state density of the susceptible class.

Livestock model

We can determine an expression for the reproductive ratio, $\mathcal{R}_0$, using the next-generation matrix approach (NGM) [Diekmann et al., 2010], where $\mathcal{R}_0$ is given by the spectral radius of the matrix $\mathbf{FV^{-1}}$ evaluated at the disease-free steady state.

$$\begin{aligned}\mathcal{R}_0 &= \rho\left(\mathbf{FV^{-1}}(S^*,0,0)\right)\\ &= \frac{N\beta+p(c_S+d)}{d+\alpha+c_I+\gamma}\end{aligned} \tag{S.11}$$

where

$$\mathbf{F} = \begin{bmatrix} \frac{p((N-I)(\alpha I+c_I I+c_R R+c_S S)+N(dN+\alpha I+c_I I))}{N^2}+\beta S & \frac{pI(I(c_R-c_S-\alpha)+S(c_R-c_S))}{N^2} \\ \frac{pR(R(\alpha+c_I-c_R)+S(\alpha+c_I-c_S))}{N^2} & \frac{p\left((S+I)(\alpha I+c_I I+2c_R R+c_S S)+c_R R^2+dN^2\right)}{N} \end{bmatrix},$$
$$\mathbf{V} = \begin{bmatrix} d+\alpha+c_I+\gamma & 0 \\ -\gamma & d+c_R+\eta \end{bmatrix}$$

and $S^* = N$ is the disease-free steady state density of the susceptible class. Note, another eigenvalue, $\lambda_2 = \frac{p(c_S+d)}{d+c_R+\eta}$, that relates to a steady state where $I = 0$, can also be found. However, for our parameter choices this will always be less than the reproductive number given in equation (S.13).

S.5 Impact of culling and evolution on wildlife and livestock management practices

In the main text we show how culling affects R_0 in the livestock model (Figure 5). In Figure S.4 we show these results for a wider set of parameters. In the main text we also show the results

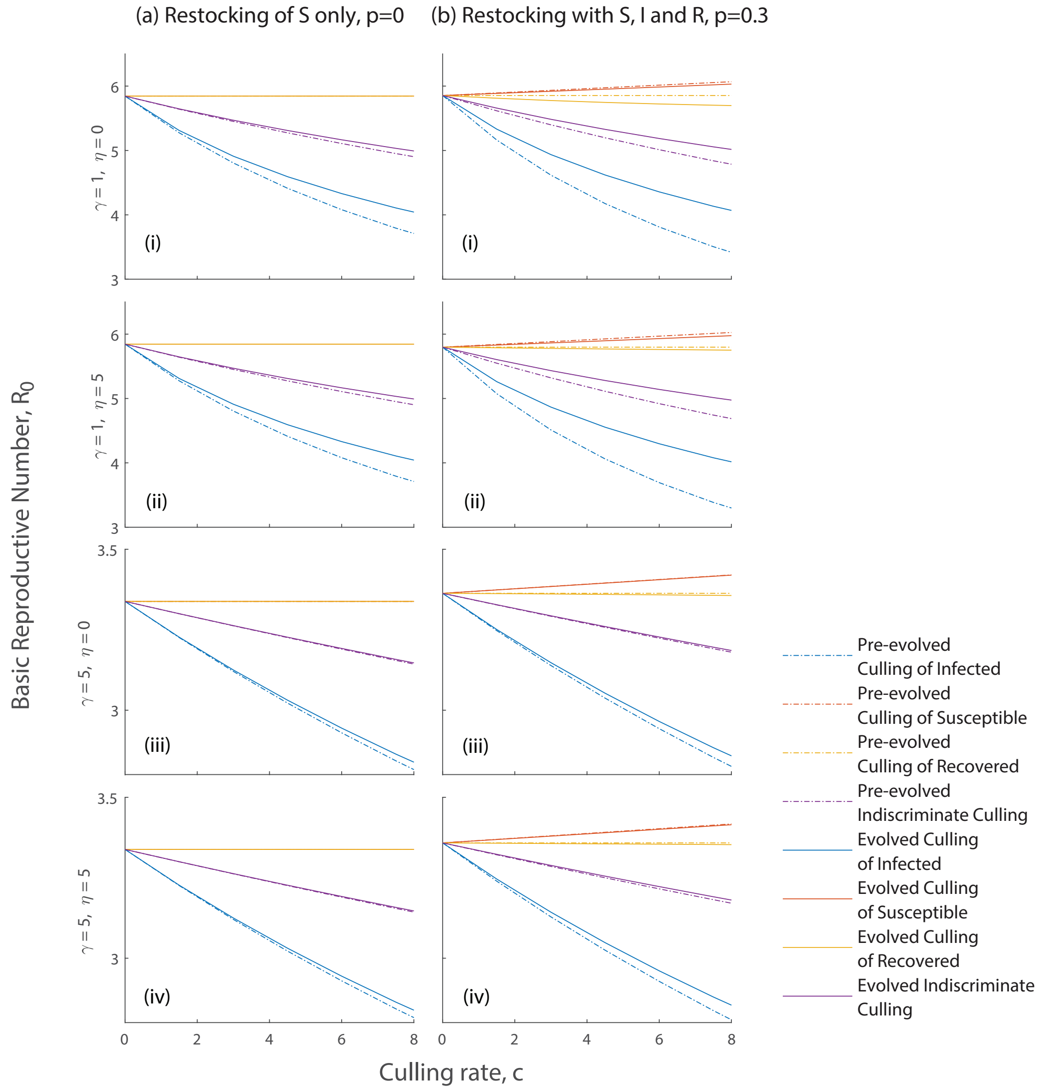

Figure S.4: Basic reproductive number, R_0, for varying rates of culling, under a livestock model framework (see equations 2). Results are shown for (a) restocking with susceptible individuals (only) and (b) restocking that includes infected and recovered individuals, $p = 0.3$, and for different infection types with (i) $\eta = 0, \gamma = 1$, (ii) $\eta = 5, \gamma = 1$, (iii) $\eta = 0, \gamma = 5$ and (iv) $\eta = 5, \gamma = 5$. Different types of culling are indicated with (blue) culling of infected individuals only, (red) culling of susceptible individuals only, (yellow) culling of recovered individuals only and (purple) indiscriminate culling. We show results when virulence evolves in response to culling (solid line) and when virulence is fixed at the pre-culling level (dot-dashed line). When not varied in the figure, parameters are taken from Tables 1 and 2 in the main text.

indicating the population decline against culling level in the wildlife model (Figure 6). In Figure S.5) we show these results for a wider set of parameters.

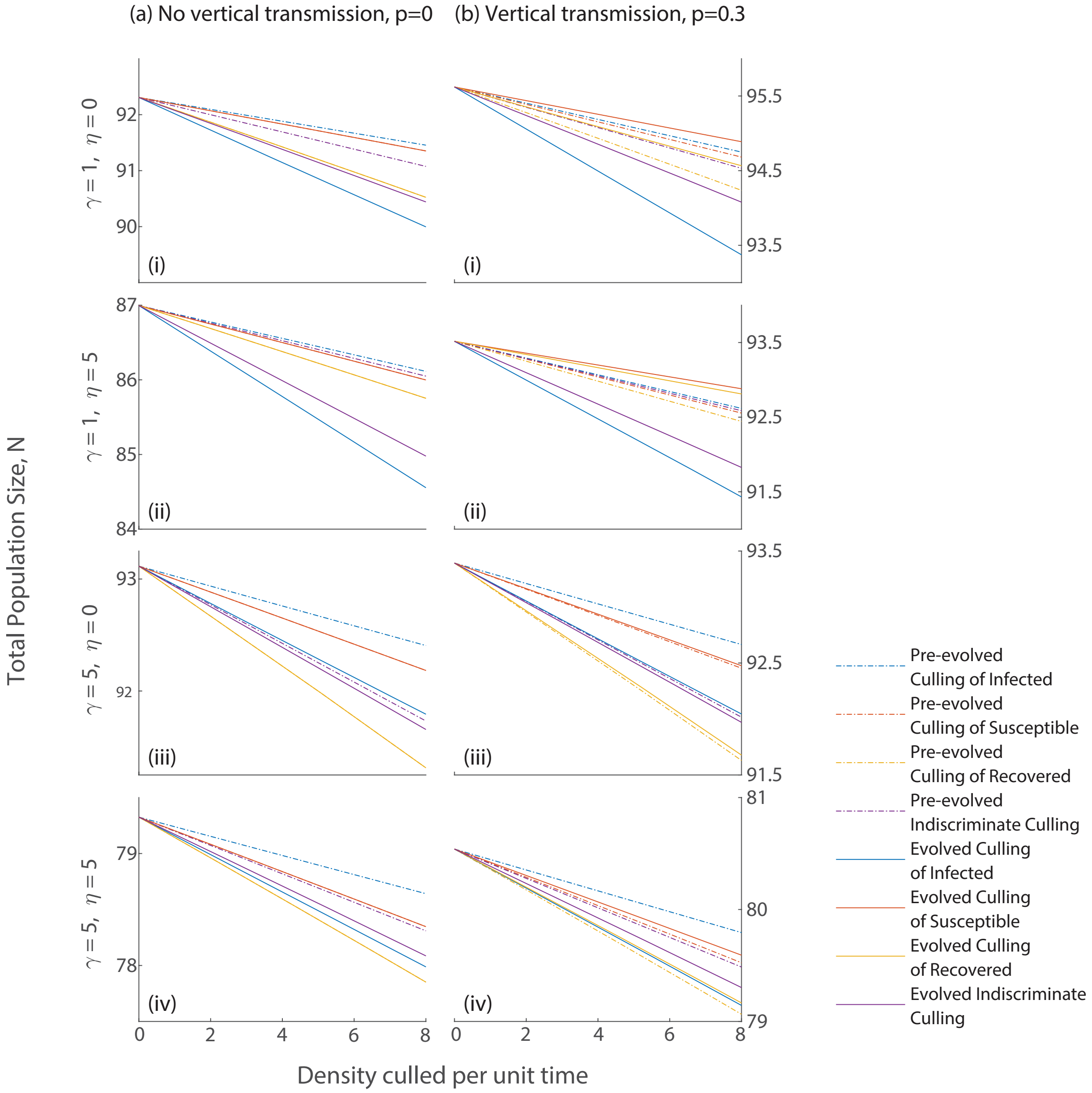

Figure S.5: The total population density, N, for varying densities culled per unit time, under a wildlife model framework (see equations 1). Results are shown in (a) the absence of vertical transmission, $p = 0$ and (b) with vertical transmission, $p = 0.3$ and for different infection types with (i) $\eta = 0, \gamma = 1$, (ii) $\eta = 5, \gamma = 1$, (iii) $\eta = 0, \gamma = 5$ and (iv) $\eta = 5, \gamma = 5$. Different types of culling are indicated with (blue) culling of infected individuals only, (red) culling of susceptible individuals only, (yellow) culling of recovered individuals only and (purple) indiscriminate culling. We show results when virulence evolves in response to culling (solid line) and when virulence is fixed at the pre-culling level (dot-dashed line). When not varied in the figure, parameters are taken from Tables 1 and 2 in the main text.

S.6 Wildlife model with frequency-dependent transmission

S.6.1 Model Formulation

We adapt the wildlife model with density-dependent infection transmission (see equations (1)), to instead have frequency-dependent infection transmission. The model is as follows:

$$\begin{aligned}
\frac{dS}{dt} &= (bS + bR + b(1-p)I)(1 - q_b N) - (d + q_d N)S - \beta S \frac{I}{N} - c_S S + \eta R, \\
\frac{dI}{dt} &= bpI(1 - q_b N) + \beta S \frac{I}{N} - ((d + q_d N) + \alpha + c_I + \gamma)I, \\
\frac{dR}{dt} &= \gamma I - (d(1 + q_d N) + c_R + \eta)R.
\end{aligned} \tag{S.12}$$

All population classes and parameters are as detailed in the main paper (for parameter definitions and values see section 2 and table 1 in the main paper). However, as the transmission is now frequency-dependent we modify the minimum and maximum values for the transmission coefficient, β, in the absence of vertical transmission ($p = 0$) and density-dependent death ($q_d = 0$), such that the evolutionarily stable virulence in both model frameworks is equivalent. These modified model parameters are given in table S.1.

S.6.2 Evolutionary Dynamics

If we assume the mutant parameters are $\bar{\beta}, \bar{\alpha}$ and the resident parameters are β, α the fitness expression for the wildlife model with frequency-dependent transmission is given by:

$$\bar{s} = \left(\bar{\beta} - \beta\right) \frac{S}{N} - (\bar{\alpha} - \alpha),$$

where S denotes the steady state density of the resident susceptible population. Given that $\beta = f(\alpha)$, from our trade-off function, we can determine the local directional gradient of the mutant fitness:

$$\frac{\partial \bar{s}}{\partial \bar{\alpha}} = f'(\bar{\alpha}) \frac{S}{N} - 1.$$

This will reach a singular strategy, α^*, when the following condition is met:

$$f'(\bar{\alpha}) = \frac{N^*}{S^*},$$

Table S.1: Parameters for the trade-off function in the wildlife frequency-dependent transmission model (see equations S.14).

Parameter	Value	Description
a	-0.5	Strength of curve for trade-off function
β_{min}	10	Minimum value for transmission coefficient β
β_{max}	50	Maximum value for transmission coefficient β
α_{min}	0	Minimum value for disease-induced mortality rate α
α_{max}	10	Maximum value for disease-induced mortality rate α

where S^* denotes the steady state value of the susceptible population evaluated at the singular strategy, α^*, and N^*, the steady state value of the total population evaluated at the singular strategy.

For an evolutionary attractor we require that the singular strategy, α^*, is both evolutionary stable (ES) and convergence stable (CS) [Bowers et al., 2005, Geritz et al., 1998, Metz et al., 1996]. We outline these conditiions for the wildlife model with frequency-dependent transmission (see equations (S.14)) below:

ES requires:

$$\left.\frac{\partial^2 \bar{s}}{\partial \bar{\alpha}^2}\right|_{\alpha=\bar{\alpha}=\alpha^*} = \left.(f''(\bar{\alpha})SI)\right|_{\alpha=\bar{\alpha}=\alpha^*} = f''(\alpha^*)\frac{S^*}{N^*} < 0. \tag{S.13}$$

CS requires:

$$\left.\left(\frac{\partial^2 \bar{s}}{\partial \alpha^2} - \frac{\partial^2 \bar{s}}{\partial \bar{\alpha}^2}\right)\right|_{\alpha=\bar{\alpha}=\alpha^*} = -2f'(\alpha^*)\left(\frac{1}{N^*}\left.\frac{\partial S}{\partial \alpha}\right|_{\alpha=\bar{\alpha}=\alpha^*} - \frac{S^*}{{N^*}^2}\left.\frac{\partial N}{\partial \alpha}\right|_{\alpha=\bar{\alpha}=\alpha^*}\right) - 2f''(\alpha^*)\frac{S^*}{N^*} > 0. \tag{S.14}$$

Here, S^* and N^* denote the steady state density of the susceptible class and total population, respectively, at the singular strategy, α^*.

S.6.3 Results

In Figure S.6 we compare the effect of vertical transmission on the evolution of virulence in a wildlife model with density-dependent or frequency-dependent transmission. In Figure S.7) we examine the impact of culling on the evolution of infection and virulence in a wildlife model with frequency-dependent transmission (which can be compared with Figure (3) in the main text that considers density-dependent transmission). Our results show that the form of infection transmission makes little difference on the evolution of infection.

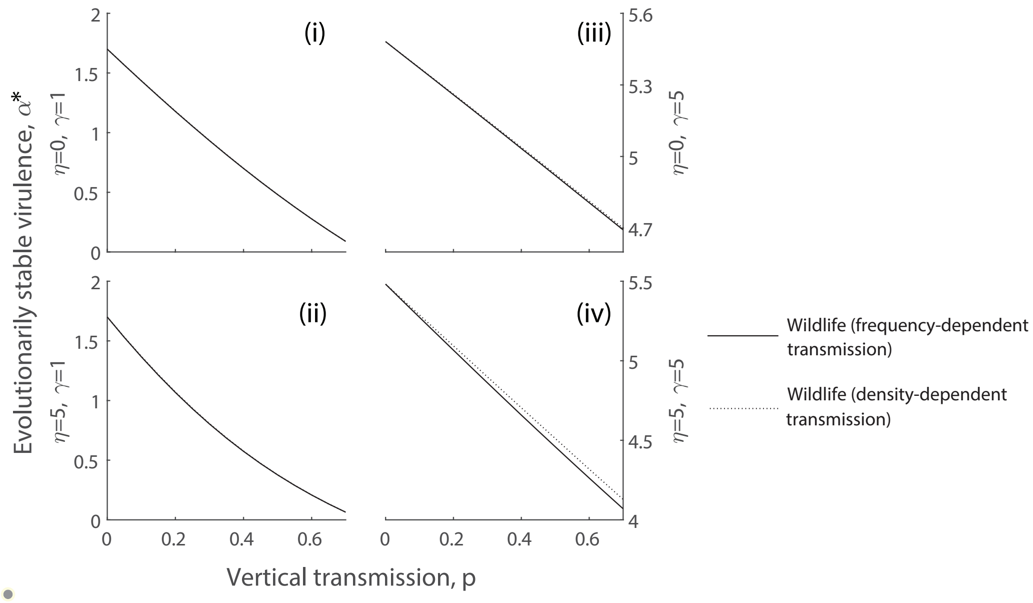

Figure S.6: Evolved level of virulence, α^*, for a varying level of vertical transmission, p, under a wildlife model with frequency-dependent transmission (see equations S.14) (solid line) and a wildlife model with density-dependent transmission (see equations 1) (dot-dashed line). Results are shown for different infection types with (i) $\eta = 0, \gamma = 1$, (ii) $\eta = 5, \gamma = 1$, (iii) $\eta = 0, \gamma = 5$ and (iv) $\eta = 5, \gamma = 5$. When not varied in the figure, parameters are taken from Tables 1, 2 and S.1 with β given by our trade-off function (equation 3). Note, for all parameter choices, aside from $\eta = \gamma = 5$, the wildlife model with frequency-dependent transmission matches that of the wildlife model with density-dependent transmission.

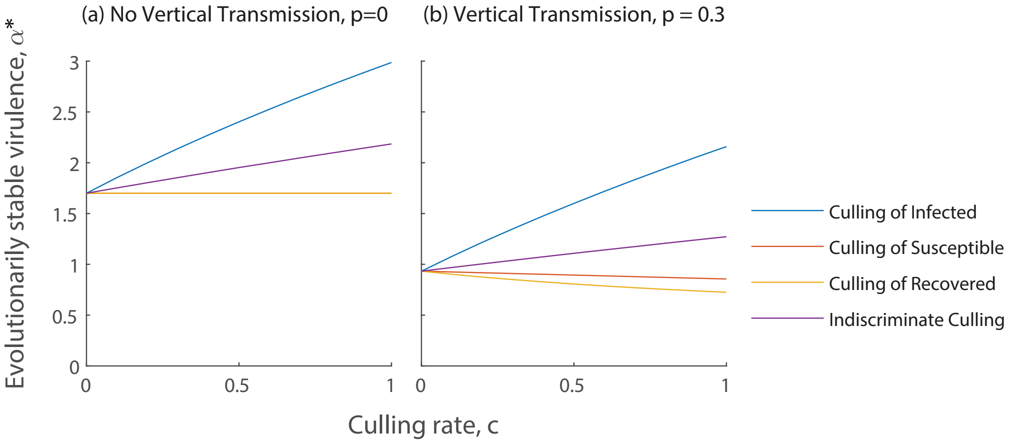

Figure S.7: Evolved level of virulence, α^*, for varying rates of culling, under a wildlife model framework with frequency-dependent transmission (see equations S.14). Results are shown in (a) the absence of vertical transmission and (b) with vertical transmission, $p = 0.3$. Different types of culling are indicated with (blue) culling of infected individuals only, (red) culling of susceptible individuals only, (yellow) culling of recovered individuals only and (purple) indiscriminate culling. When not varied in the figure, parameters are taken from Tables 1 and S.1, with $\gamma = 1$ and $\eta = 0$.

. . . .

References

[Bowers et al., 2005] Bowers, R., Hoyle, A., White, A., and Boots, M. (2005). The geometric theory of adaptive evolution: trade-off and invasion plots. *Journal of Theoretical Biology*, 233(3):363–377.

[Diekmann et al., 2010] Diekmann, O., Heesterbeek, J., and Roberts, M. (2010). The construction of next-generation matrices for compartmental epidemic models. *J.R.Soc.Interface*, 7:873–885.

[Geritz et al., 1998] Geritz, S., Kisdi, E., Meszéna, G., and Metz, J. (1998). Evolutionary singular strategies and the adaptive growth and branching of the evolutionary tree. *Evol. Ecol.*, 12:35–57.

[Metz et al., 1996] Metz, J., Geritz, S., Meszena, G., Jacobs, F., and Van HeerWaarden, J. (1996). Adaptive dynamics: a geometric study of the consequences of nearly faithful reproduction. In Van Strien, S. and Verduyn Lunel, S., editors, *Stochastic and Spatial Structures of Dynamical Systems*, pages 183–231. North-Holland.
